# Alterations of lower respiratory tract microbiome and short-chain fatty acids in different segments in lung cancer: a multiomics analysis

**DOI:** 10.3389/fcimb.2023.1261284

**Published:** 2023-10-16

**Authors:** Yong Zhang, Xiangxiang Chen, Yuan Wang, Ling Li, Qing Ju, Yan Zhang, Hangtian Xi, Fahan Wang, Dan Qiu, Xingchen Liu, Ning Chang, Weiqi Zhang, Cong Zhang, Ke Wang, Ling Li, Jian Zhang

**Affiliations:** ^1^ Department of Pulmonary and Critical Care of Medicine, The First Affiliated Hospital of Fourth Military Medical University, Xi’an, China; ^2^ National Translational Science Center for Molecular Medicine & Department of Cell Biology, Fourth Military Medical University, Xi’an, China; ^3^ Department of Microbiology, School of Basic Medicine of Fourth Military Medical University, Xi’an, China; ^4^ Department of Pediatrics, The First Affiliated Hospital of Fourth Military Medical University, Xi’an, China; ^5^ School of Basic Medicine, Fourth Military Medical University, Xi’an, China; ^6^ Department of Radiology, The First Affiliated Hospital of Fourth Military Medical University, Xi’an, China; ^7^ Department of Radiation Oncology, The First Affiliated Hospital of Fourth Military Medical University, Xi’an, China

**Keywords:** lung cancer, lower respiratory tract microbiome, metagenomic sequencing, short chain fatty acids, machine learning

## Abstract

**Introduction:**

The lower respiratory tract microbiome is widely studied to pinpoint microbial dysbiosis of diversity or abundance that is linked to a number of chronic respiratory illnesses. However, it is vital to clarify how the microbiome, through the release of microbial metabolites, impacts lung health and oncogenesis.

**Methods:**

In order to discover the powerful correlations between microbial metabolites and disease, we collected, under electronic bronchoscopy examinations, samples of paired bronchoalveolar lavage fluids (BALFs) from tumor-burden lung segments and ipsilateral non-tumor sites from 28 lung cancer participants, further performing metagenomic sequencing, short-chain fatty acid (SCFA) metabolomics, and multiomics analysis to uncover the potential correlations of the microbiome and SCFAs in lung cancer.

**Results:**

In comparison to BALFs from normal lung segments of the same participant, those from lung cancer burden lung segments had slightly decreased microbial diversity in the lower respiratory tract. With 18 differentially prevalent microbial species, including the well-known carcinogens *Campylobacter jejuni* and *Nesseria polysaccharea*, the relative species abundance in the lower respiratory tract microbiome did not significantly differ between the two groups. Additionally, a collection of commonly recognized probiotic metabolites called short-chain fatty acids showed little significance in either group independently but revealed a strong predictive value when using an integrated model by machine learning. Multiomics also discovered particular species related to SCFAs, showing a positive correlation with *Brachyspira hydrosenteriae* and a negative one with *Pseudomonas* at the genus level, despite limited detection in lower airways. Of note, these distinct microbiota and metabolites corresponded with clinical traits that still required confirmation.

**Conclusions:**

Further analysis of metagenome functional capacity revealed that genes encoding environmental information processing and metabolism pathways were enriched in the lower respiratory tract metagenomes of lung cancer patients, further supporting the oncogenesis function of various microbial species by different metabolites. These findings point to a potent relationship between particular components of the integrated microbiota-metabolites network and lung cancer, with implications for screening and diagnosis in clinical settings.

## Introduction

A growing body of evidence implies that perturbations of the compositions within the human microbiome exert great influence on a broad array of human diseases, including a set of cancer types (Cullin et al., 2021; Sepich-Poore et al., 2021; Yang et al., 2023). As a widely accepted perspective, gut microbiota, due to vast microbial coverage and quantity within the digestive tract, is confirmed to shed bidirectional light on lung cancer by crosstalk between microbiota and host cells (Liu et al., 2019; Dong et al., 2021; Dohlman et al., 2022). Compared with remote modulation by gut microbiome-released metabolites, microbiota in local pulmonary microecological environments, which were previously considered to be sterile, is gradually receiving widespread attention in oncogenesis, development, and drug resistance of lung cancer (Routy et al., 2018; Tsay et al., 2018; Patnaik et al., 2021; Zitvogel and Kroemer, 2021). Importantly, colonization of microbes in the lungs, especially those in the lower respiratory tract, features much lower bacterial biomass but higher relative diversity, which may be reversed with elevated bioburden and descending bacterial diversity followed by several taxa in a significant proportion in suppurative and infectious diseases (Lanaspa et al., 2017; Man et al., 2017; Singh et al., 2017). However, only limited research focused on the potential role of the lower respiratory tract microbiome in the initiation and development of lung cancer and further studies are still needed for a detailed exploration of this.

Analysis of the lower respiratory tract microbiome is still intractable, partially due to the complexity of sample detection and the low biomass planted in the local respiratory tract, impeding the accuracy and sensitivity of bacterial community processing and sequencing (Drengenes et al., 2019). Different from conventional 16S rRNA gene sequencing, metagenomics seems more effective in eliminating latent hosted and operational contamination, making it an alternative to further uncover the microbial composition of the lower respiratory tract microbiome (Kurian et al., 2020; Fromentin et al., 2021; Lamoureux et al., 2022). Of note, although characterized with significantly lower bacterial communities than those detected by oropharyngeal swabs or washes, sputum samples, and bronchial aspirates from the upper airway, bronchoalveolar lavage fluids (BALFs) are usually given preference to sequence lower respiratory tract microbiome and their metabolites (Glendinning et al., 2017; Tsang et al., 2021).

Short-chain fatty acids (SCFAs), which are chemically composed of a carboxylic acid moiety and a small hydrocarbon chain under six including acetic, propionic, and butyric acids, are a subset of intermediate fatty acid metabolites mainly produced by anaerobic bacteria in the intestinal tract during the fermentation of fibers and dietary carbohydrates. SCFAs perform a beneficial function in the maintenance of health and in guarding against cancers (Sivaprakasam et al., 2016; Mirzaei et al., 2021; Van Der Hee and Wells, 2021). Mechanically, SCFAs are known to modify extensive cellular processes by direct activation of G protein-coupled receptors (GPCRs) (Kim et al., 2013), inhibition of histone deacetylases (HDACs) (Shen et al., 2017), and stabilization of the hypoxia-inducible factor (HIF) signaling pathway (Shen et al., 2017) in a ligand-receptor interaction by regulating epithelial homeostasis and stimulating anti-tumor immune activity (Trompette et al., 2014; Kim et al., 2016; Zou et al., 2018; Matsushita et al., 2021). Intriguingly, with the further exploration of the microbiome in a liquid layer on the surface of the respiratory tract and alveoli, it has been observed that lower respiratory tract-derived SCFAs might also be involved in the modulation of the host metabolism and immunity homeostasis. The inhibitory function of SCFAs on lung cancer deserves additional attention.

In order to address the correlation of lower respiratory tract microbiome and SCFAs, as well as their potential interaction with lung cancer, we investigated the microbial communities and SCFAs of the lower respiratory tract by metagenomic and targeted metabolome sequencing in BALF from tumor-burden lung segments and ipsilateral non-tumor sites of the same lung cancer patients. Employing an in-depth multiomics combined analysis, we aimed to validate the predictive role of SCFAs and specific microbiota in tumorigenesis and their predictive effects in the diagnosis and prevention of lung cancer in clinical practice.

## Materials and methods

### Study design and participant recruitment

The study cohort consisted of a subset of hospitalized subjects enrolled in our Clinical Humoral Biological Sample Library. We collected 128 cases that, according to their CT scanning characteristics, were suspected lung cancer (LC) cases, and excluded the inappropriate patients in light of our clinical research design (#2021LC2115). Details of inclusion and exclusion criteria and workflow are displayed in **Table 1**; **Figure S1**. A final diagnosis of LC depended on pathological characteristics of tissue samples from electronic bronchoscopy-mediated needle aspiration biopsy after BALF collection. At enrollment, we included patients with lung cancer who had not been treated with pharmacological interventions for the previous 3 months, such as anti-tumor regimes, antibiotics, probiotics intake, and other potential preparations that might affect local and extensive microbial compositions. Exclusion criteria included patients with concomitant infectious or inflammatory respiratory diseases, tumor-associated obstructive pneumonia, and patients using glucocorticoid drugs in the preceding 6 months. All patients fully understood the objectives and were volunteers for potential inspection risks. Each subject signed an informed consent approved by the Ethics Committee of the First Affiliated Hospital of the Air Force Medical University; the Academic Integrity Supervision Committee of Air Force Military Medical University carried out supervision of the whole course within the study.

**Table 1:** Demographic and clinical characteristics of the cohort.

Variable	Number (Mean ± SD or %)
Age (yrs)	63.59 ± 8.95
Sex (male,%)	20 (74.07)
BMI (kg/m^2^)	23.12 ± 2.35
Smoking status (Yes,%)	15 (55.56)
Pathological types (%)	
Adenocarcinoma	12 (44.44)
Squamouscarcinoma	9 (33.33)
Small cell lung cancer	5 (18.52)
Others	1 (3.70)
Mutations (%)	
EGFR	7 (25.93)
Others	2 (7.40)
None	18 (66.67)
Clinical stages (%)	
I	0 (0)
II	2 (7.41)
III	2 (7.41)
IV	16 (59.25)
Unknown	7 (25.93)

### Sample collection and preservation

Samples processed for microbiota analysis were collected from patients consulting for medical assistance in our center who needed electronic bronchoscopy-mediated needle aspiration biopsy to reach a definite diagnosis. Before that, bronchial and alveolar lavage fluid was obtained from normal lung segment (NLS) and tumor-burden lung segments (TBLS) successively within the same lung lobe. Each lavage was treated with preheated sterile physiological saline for 50-60ml, maintaining a stable recovery rate of >60%. All samples intended for microbial analysis were under centrifugation at 4°C 12000rpm for 40 min. Centrifugal sedimentation and supernatant were segregated and restored at -80°C for microbial and targeted metabolomics analysis concurrently until processing. All processes strictly abided by sterile operating standards.

### DNA isolation and shotgun metagenomics sequencing

BALF precipitation samples (1-3mg) were weighed in 2 ml microcentrifuge tubes and placed on ice. Total DNA from the lower respiratory tract microbiotas was extracted using the QIAamp Fast DNA Stool Mini Kit (QIAGEN, Germany) per the manufacturer’s instructions (see the QIAamp Fast DNA Stool Mini Kit Handbook, www.qiagen.com/handbooks). The degradation degree and potential contamination of the DNA were analyzed using 1% agarose gels. The DNA purity was determined using the NanoPhotometer^®^ spectrophotometer (IMPLEN, CA, USA). DNA samples were further diluted with sterile water to an OD value between 1.8 and 2.0, measuring with the Qubit^®^ dsDNA Assay Kit in Qubit^®^ 2.0 Fluorometer (Life Technologies, CA, USA). One microgram of qualified DNA was used to construct the library via NEBNext^®^ Ultra DNA Library Prep Kit for Illumina (NEB, USA). DNA samples were fragmented to 350 bp by sonication, and then the DNA fragments were end-polished, A-tailed, and ligated with the full-length adaptor for Illumina sequencing with further PCR amplification. Libraries were analyzed for size distribution using the Agilent2100 Bioanalyzer (Agilent, USA) and quantified via real-time PCR to keep size distribution of DNA fragments >3nM. The libraries were then sequenced on an Illumina PE150 HiSeq platform.

### Preprocessing of sequencing results and metagenomic assembly

Raw data obtained from the Illumina PE150 sequencing platform were preprocessed by Readfq (V8, https://github.com/cjfields/readfq) to obtain clean data for subsequent analysis. The clean data were utilized for assembly analysis with MEGAHIT software (v1.0.4-beta in a –presets meta-large (–end-to-end, –sensitive, -I 200, -X 400) parameter settings, and the Scaftigs were obtained by breaking the resulted scaffolds from the N junction. All the sample details on the quality of their assemblies are present in **Table S1**.

### Gene prediction and abundance analysis

The Scaftigs (≥ 500 bp) were submitted to predict the open reading frame (ORF) using MetaGeneMark (V2.10; http://topaz.gatech.edu/GeneMark/) to filter out the excessive information with a length less than 100nt, and CD-HIT software (V4.5.8; http://www.bioinformatics.org/cd-hit/) to eliminate redundancy. Clean data of each sample was aligned to the initial gene catalog by using Bowtie2 (V2.2.4; https://bowtie-bio.sourceforge.net/bowtie2/) to calculate the number of reads of the genes on each sample alignment, with parameter settings: –end-to-end, –sensitive, -I 200, -x 400. Genes with reads ≤2 in each sample were filtered out to finally determine the gene catalog (Unigenes) for subsequent analysis (**Tables S2, S3**). Based on the number of reads aligned and the length of the gene, the abundance of each gene in each sample was calculated by the following formula:


$$G_{k}-\frac{r_{k}}{L_{k}}\times \frac{1}{\sum {\;}_{i=1}^{n}\frac{r_{i}}{L_{i}}}$$

in which r is the number of gene reads on alignment, and L is the length of the gene (Qin et al., 2010). Based on the abundance of each gene in the gene catalog in each sample, basic information statistics, core-pan gene analysis, correlation analysis between samples, and Venn diagram analysis of gene number were performed.

### Species annotation

The obtained unigenes were used to blast the sequences for the bacteria, fungi, archaea, and viruses, which were extracted from the NR database (V20180102; https://www.ncbi.nlm.nih.gov/) of NCBI using DIAMOND software (V0.9.9.110; https://github.com/bbuchfink/diamond/). We used the lowest common ancestor (LCA) algorithm to obtain the number of genes and abundance information for each sample in each taxonomic hierarchy (kingdom, phylum, class, order, family, genus, and species). DIAMOND software was also used to blast unigenes to functional databases, including the KEGG (V20180101; http://www.kegg.jp/kegg/) databases, for the blast results, and the best blast hit was used for subsequent analysis.

### Advanced analysis of metagenomic data

According to the alignment results, the relative abundance at different functional levels was calculated (the relative abundance at each functional level was equal to the sum of the relative abundance of genes annotated at that functional level). The gene number table of each sample at each taxonomy level was derived from the result of functional annotation and gene abundance table. The number of genes with a certain function in a sample was equal to the number of genes whose abundance was non-zero among the genes annotated with this function. Based on the abundance table at each taxonomy level, annotated genes statistics, relative abundance overview, and abundance clustering heat map were carried out, combined with PCA and NMDS analysis of dimension reduction, ANOSIM analysis of inter-/intra-group differences based on functional abundance, metabolic pathway comparative analysis, as well as Metastat and LEfSe analysis on the inter-group functional difference.

### Quantification of BALF metabolites

SCFA contents in BALF supernatant were detected by Metware Biotechnology Co., Ltd. (Wuhan, China) with gas chromatography-tandem mass spectrometry analysis. Briefly, BALF samples were thawed and vortexed for 1 min prior to analysis. A total of 50μL of samples were mixed with 100μL of phosphoric acid (0.5% v/v) solution, vertexing for 3 min and ultrasonicating for 5 min. After that, the mixture was centrifuged at 12000 rpm for 10 min at a temperature of 4°C. The supernatant was collected and used for GC-MS/MS analysis. Agilent 7890B gas chromatograph coupled to a 7000D mass spectrometer with a DB-5MS column (30m length × 0.25mm inner diameter × 0.25μm film thickness; J&W Scientific, Folsom, CA) was used. Helium was used as the carrier gas, at a flow rate of 1.2mL/min. Injections were made in the spitless mode, and the injection volume was 2μL. The oven temperature was held at 90°C for 1 min, raised to 100°C at a rate of 25°C/min, raised to 150°C at a rate of 20°C/min, and held at 150°C for 0.6 min. Then, the temperature was further raised to 200°C at a rate of 25°C/min and held at 200°C for 0.5 min. After running for 3 min, all samples were analyzed in multiple reaction monitoring mode. The temperature of the injector inlet and transfer line were held at 200°C and 230°C, respectively.

### Random forest and machine learning prediction models

The random forest algorithm was applied to elucidate the influence of candidates on lung cancer prediction by repeated cross-validation. Further analyses were carried out in R software (v3.5.2). The LASSO logistic regression model was performed to select the most useful prognostic risk factors for SCFA candidates in BALFs collected from lower respiratory tracts. All samples were identified using dummy variables. We used R software version 3.6.1 and the “glmnet” package (R Foundation for Statistical Computing, Vienna, Austria) to perform the LASSO logistic regression analysis.

### Statistical analysis

The significance of the differences between groups was analyzed using the Wilcoxon rank-sum test and ANOSIM with *P* value< 0.05 (5% level of probability) with VEGAN of R package being considered to be significant and denoted as follows: ^*^ *P*<0.05, ^**^ *P*<0.01, and^***^ *P*<0.001. The statistical significance was adjusted for multiple testing using FDR correction with the cutoff adjusted p-value< 0.05 unless otherwise stated. The receiver operating characteristic curve (ROC) analysis was performed using the R project, and the discriminative power of the predictor was assessed by calculating the area under the receiver operating characteristic curves (AUC). A variable with an AUC above 0.7 was considered useful. Significant differences between corresponding subgroups were determined via an unpaired t-test and a false discovery rate approach using the two-stage linear step-up procedure with a false discovery rate (Q) of 1%. Testing conditions were analyzed individually, without assuming a consistent SD. Statistical analysis was performed with GraphPad Prism (V9.0.0 for Windows; www.graphpad.com).

## Results

### Study group enrollment and clinical characteristics

**Figure 1:**
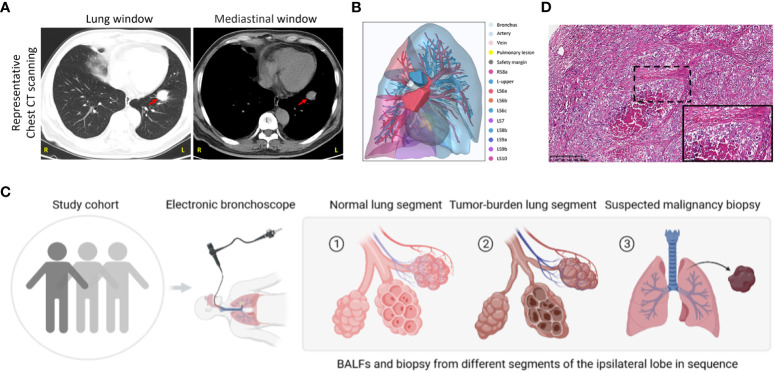
Study group enrollment and clinical characteristics. **(A)** Chest CT scan images in lung and mediastinal windows of a representative patient in the same slice. Red arrow, suspected malignant lesion. R, right; L, left. **(B)** 3D reconstruction of lung lesions within the vulnerable segment. Indicated annotations are listed on the right. **(C)** Sample collection scheme and corresponding processes. **(D)** Representative images of HE staining in the patient mentioned above. Scale bar, 200μm (10×) and 50μm (40×, inset).

From May 2022 to December 2022, we collected 128 patients with highly suspected lung cancer based on computed tomography scanning (CT) with typical malignant imaging features, including solitary or multifocal mass nodular shadow, unsmooth edges with a burr, and microvascular insertion, in light of independent judgment from our Pulmonary Nodule Diagnosis and Treatment Center. Typical CT scanning and corresponding 3D view of the targeted lesion within a representative patient among this cohort was displayed as follows (**Figures 1A, B**). All subjects were evaluated to undergo lung malignant lesion biopsy after bronchoalveolar lavage in adjacent normal segments of the ipsilateral lobe and tumor-burden lung segment via electronic bronchoscope (**Figure 1C**). After the exclusion of benign lesions and other interference factors of sample acquisition, only those patients with pathological diagnoses of malignancy were successfully enrolled, with follow-up sequencing and analysis being carried out (**Figure 1D**). The demographics of the participants are shown in **Table 1** and specific inclusion criteria and other exclusion criteria are displayed as a flowchart in **Figure S1**. Since the samples were also taken as the self-control of the same patient, we did not set up a blank control group in this study.

### Lower respiratory tract microbiome diversity decreased in tumor-burden segments

**Figure 2:**
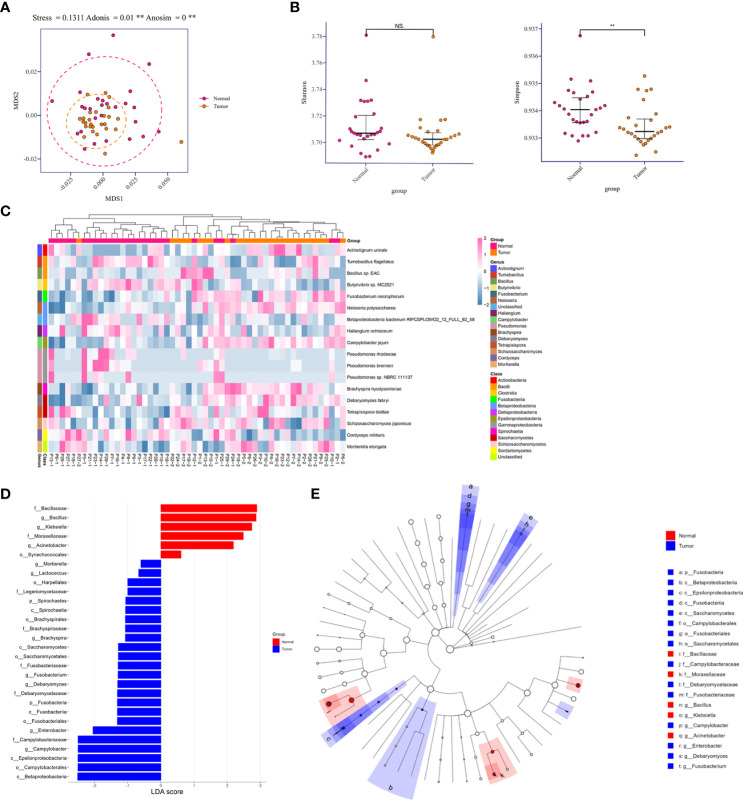
Relative abundances in lower respiratory tract microbiome and comparison of diversity analysis. **(A)** MDS plot of normal and tumor-burden lung segments in the same lung cancer patients based on the lower respiratory tract microbial compositions using Bray-Curtis dissimilarities (Stress=0.1311; ADONIS *P*=0.001; ANOSIM *P*<0.001). Intra-patient samples are linked to each other. **(B)** Alpha-diversity comparison of indicated groups by the Shannon index (No significance, Wilcoxon rank sum test) and Simpson index (^**^ *P*<0.001, Wilcoxon rank sum test). **(C)** Heatmap of differentially abundant species detected in the comparison of two groups within each sample. **(D)** Distribution diagram of the LDA score in both groups and results of the LEfSe analysis based on the LDA score to screen the candidate biomarkers. **(E)** Cladogram based on different candidates from **(D)**. The red and blue nodes represent the microorganisms that mattered most in each group. MDS, multidimensional scaling. Normal, normal lung segments; Tumor, tumor-burden lung segments. NS, no significance.

To determine compositional diversity between tumor-burden lung segment (TBLS) and ipsilateral normal lung segment (NLS), we drafted those precipitations of BALF samples collected from corresponding pulmonary segments or subsegments profiling with shotgun metagenomics sequencing, generating 1.3Gbp of sequencing data on average, and further analyzed their alpha diversity indices for the subset of final enrolled samples. Consequently, multidimensional scaling (MDS), an ordination plot based on Bray-Curtis dissimilarities, revealed distinct lower respiratory tract microbial compositions among both groups at the species level (Stress=0.1311; ADONIS *P* ^**^=0.001; ANOSIM *P* ^**^<0.001), with the majority of TBLS samples overlapping with the NLS subjects (**Figure 2A**). Additionally, the alpha-diversity comparison of indicated groups also demonstrated low taxonomic abundance in the TBLS-BALF subgroup by Simpson index (^**^ *P*<0.001, Wilcoxon rank sum test), which had no significance in the Shannon index (**Figure 2B**). Across the board, however, the lower respiratory tract microbiome at both the phylum and genus levels rarely fluctuated no matter which samples we sequenced (**Figures S2A, B**, **2E, F**). Other beta diversity analyses seemed to reach the same conclusion as mentioned above (**Figures S2C, D**). These results suggested a perspective that despite restricted loaded biomass, minor alterations in the lower respiratory tract microbiota, especially several key species, facilitated a microbiota prone to oncogenesis and tumor development.

To further explore the differences among species that presented spatially in NLS and TBLS at the time of microscopic examination, we identified 18 differentially abundant microbial species in the comparison between both groups (FDR *P*<0.05, Wilcoxon rank-sum test) (**Figure 2C**). Meanwhile, linear discriminant analysis effect size (LefSe) was performed to uncover the potential the tumor-related species biomarkers. We compared the microbiota compositions of the above candidates by the LDA score of the species (log10) to enlighten the distribution diagram of species differences (**Figure 2D**), finding that the relatively abundant microbial species were differentiated in TBLS and NLS (**Figure S3A**). CIRCOS plot of taxonomic abundance within each sample also verified the outcomes mentioned above (**Figure S2G**). A species co-abundance network among this differential genus between both lung segments further suggested that the high abundance of *C. jejuni* in TBLS might promote the dominance of *Firmicutes* and impede *Bacillota* by their intra-phylum positive associations along with the negative associations with *Bacillota* species (**Figure S3B**). Particularly, a Cladogram based on differential candidates also revealed that specific taxa related to lung cancer differed from those in normal lung segments, characterized by genus enrichment of *Campylobacter*, *Enterobacter*, *Debaryomyces*, and *Fusobacterium* in tumor-burden lung segments, which were replaced by *Bacillus*, *Klebsiella*, and *Acinetobacter* in normal lung segments (**Figure 2E** and **Table S4**), indicating the consistency of pathogenic microbial genus from biological evolutional perspectives. Collectively, these results further illustrated that compositional variations existed in cancer-loaded segments, some of which were quite distinct from those in healthy lower respiratory tract. Given the transient and significantly variable nature of normal lung microbiota in a relatively open environment (Dickson et al., 2015), the presence of a specific community could signal an ongoing pathological process providing bacteria with nutrients, a process that also deserves additional attention.

### Conjoint predictive value of multicomponent SCFAs in tumoral associations

**Figure 3:**
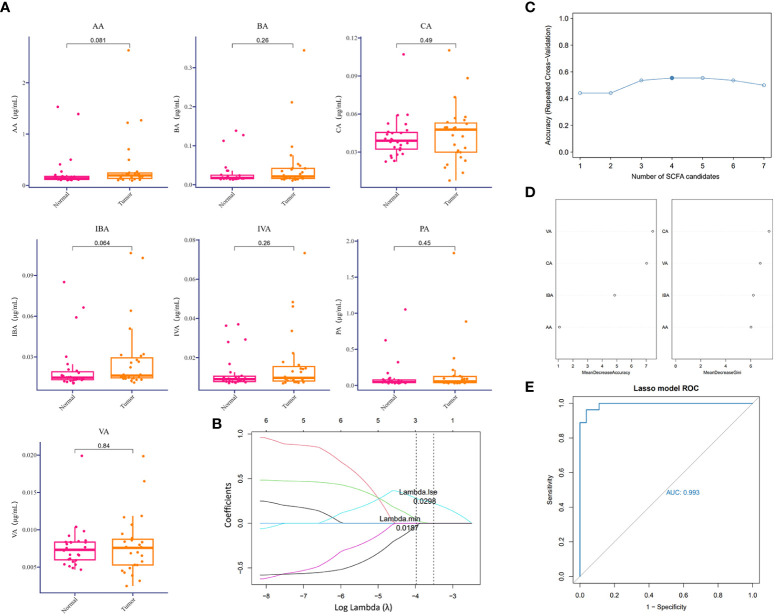
Difference analysis of SCFAs in the lower respiratory tract. **(A)** Relative detection (μg/ml) of indicated SCFAs in BALF samples collected from corresponding groups. P values are listed on each histogram. **(B)** LASSO regression coefficient profiles of the seven variables within SCFAs. Each line represents a variable. Lambda.min, the vertical dotted line at 3; Lambda.1se, the vertical dotted line at 2. **(C)** Accuracy of random forest prediction model based on repeated cross-validation from SCFA candidates. **(D)** Variable importance ranking in the effective SCFAs random forest prediction model with Mean Decrease Accuracy and Gini, respectively. LASSO, least absolute shrinkage, and selection operator. **(E)** ROC curve of SCFA-based LASSO predictive model, AUC=0.993.

Except for the direct cytotoxic effects of the majority of viruses and quite limited bacteria species, metabolites accounted for the interaction between microorganisms and hosts (Bhatt et al., 2017; Sepich-Poore et al., 2021). Short-chain fatty acids derived from the intestine are important protective lipid metabolites released by anaerobic or facultative anaerobic microbiomes to regulate distant primary tumors (Kim et al., 2016). Despite the extensive literature on the inhibitory function of gut microbiome-derived SCFAs, several lower respiratory tract microbiota at the distal end of the tumor lesion could utilize SCFAs to regulate the local ecological environment (Jin et al., 2018; Yue et al., 2020). Correspondingly, to examine the dominant SCFAs in lung cancer blockade, except for the influences from the gut microbiome, we further detected SCFAs in BALF samples mentioned above to screen out the predictive components of SCFAs in lung cancer initiation or those associated with clinical diagnosis. To our surprise, SCFAs were generally expressed at a low level in the lower respiratory tract and were slightly increased in the TBLS group but with no significance (**Figure 3A**). This outcome seemed difficult to confront in light of the probiotic effects of SCFAs in preventing tumor process, and inevitable bias or other unknown correlated noise could have contributed to the outcome. If anything, the release of SCFAs-oriented from the lower respiratory tract within different lung segments of the same lung cancer patient was prone to be identical, regardless of the differentiated microbial composition, which was in line with previous studies.

From a practical perspective, however, exploring the predictive value of a single metabolite under sophisticated circumstances in lower airways seemed unacceptable, due to the potent interactional multiplicities between the microbiome and the host. Thus, we reconstructed a machine learning-based multivariate prediction model to clarify the predictive function of SCFAs. LASSO regression coefficient profiles of the seven SCFA candidates showed that priorities for prediction were given to combined metabolites of three SCFAs, namely, CA, VA, and IBA (**Figures 3B**, **S4A**), which was also confirmed by the Random forest prediction model and ROC curve based on repeated cross-validation from SCFA candidates (**Figures 3C–E**, **S4B**). Despite restricted accuracy of under 50%, the predictive value of this combined model should be highlighted, probably because it presented a new lung cancer diagnostic approach based on metabolic exhalation detection, deserving further validation in clinical settings.

### Metagenomic and targeted metabolomic analysis with clinical characteristics

**Figure 4:**
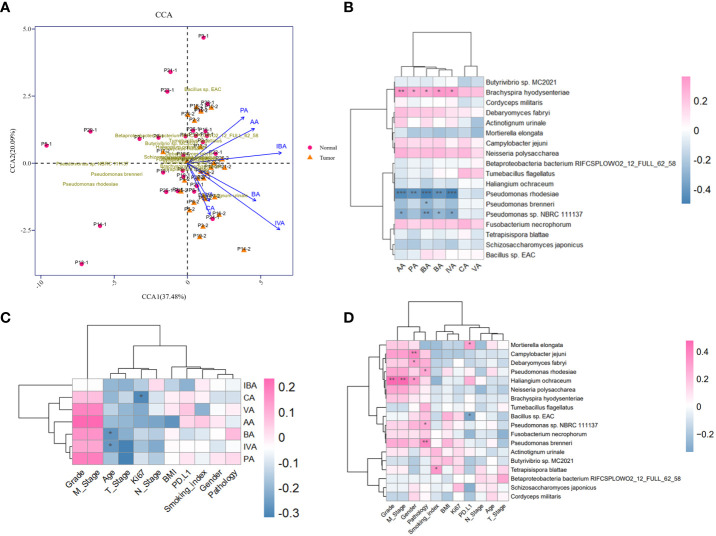
Metagenomic and metabolomic combined analysis and indicated correlation with clinical characteristics. **(A)** CCA biplot of the candidate microbial species and SCFAs. Each microbial sample is marked in the plot. **(B)** Heatmap of the correlation between candidate microbial species and SCFAs. **(C, D)** Heatmaps of potent correlation between candidate microbial species, SCFAs, and clinical information of enrolled cohort, respectively. ^*^ *P*<0.05, ^**^ *P*<0.01, and^***^ *P*<0.001. CCA, Canonical correspondence analysis.

The production of SCFAs bears a tight correlation with anaerobic or facultative anaerobic microbiome in guts, supported by sufficient findings that the fluctuation of microbial metabolites may be attributed to microbiome compositional diversity (Asnicar et al., 2021). Next, we implemented an integrated analysis of the candidate microbial species and SCFAs, in order to screen out dominant SCFA-associated microbes in tumor-burden lower respiratory tract. As a consequence, CCA profiling showed that the potential correlation between SCFAs and differential microbes mattered in tumor-burden segments with merely low efficiency (**Figure 4A**), partially due to restricted abundance and sample capacity. Heatmap of microbial species and SCFAs might present explicit correlations of differential microbes and SCFAs (**Figure 4B**), indicating a positive SCFA correlation with *Brachyspira hydysenteriae* and a negative connection with *Pseudomonas* at the genus level. These results further illustrated that the microbial-metabolic prediction model facilitated cancer screening and diagnosis by bronchoscopy-dependent BALF examination, which still deserves detailed evaluation in a large-scale population. Furthermore, as to significant correlations with clinical characteristics mentioned in other studies (Ubachs et al., 2021), we found that SCFAs and differential microbes were bound up with various clinical factors (**Figures 4C, D**), including sex, smoking status, TNM stages, and tumor gradings, although these correlations might be triggered indirectly by other unverified factors. Owing to the lack of experimental verifications of indicated candidates correlated with these characteristics, additional preferences should be given in clinical studies to further demonstrate the underlying role of the lower respiratory tract microbiome.

### Microbial metabolite-mediated host cell signaling activated in TBLS

**Figure 5:**
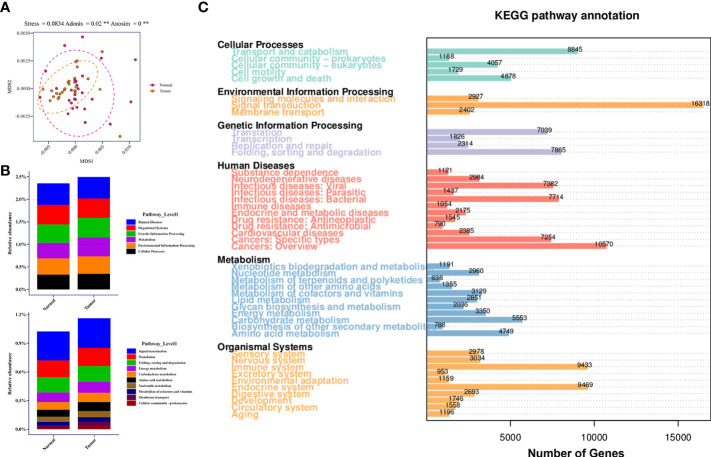
Relative abundance of KEGG pathway in lower respiratory tract microbiome. **(A)** MDS plot of samples based on KEGG pathway abundances using Bray-Curtis dissimilarities (Stress=0.0834; ADONIS *P*=0.02; ANOSIM *P*<0.001). **(B)** Relative abundance of candidate pathways at Level 1 and Level 2 in healthy and tumor-burden lung segments, respectively. **(C)** Distribution of differentially abundant KEGG pathways (FDR, Wilcoxon rank-sum test) detected in the comparison of corresponding samples. ** means P<0.01.

The Bray-Curtis dissimilarities based on KEGG pathway abundances illustrated the marginally separate clusters of NLS and TBLS (ANOSIM, ^**^ *P*<0.01) (**Figure 5A**). The KEGG pathway enrichment analysis of the metagenomic data showed that activated pathways in TBLS overlapped with those in NLS, whereas minor differences were detected only in environmental information processing and metabolism-related cascades, including cellular community-prokaryotes, signaling transduction, membrane transport, metabolism of cofactors and vitamins, and carbohydrate metabolism (**Figures 5B**, **S5A, B**). It is reasonable to speculate that microbe-mediated host interactions were achieved by microbial metabolites, which might induce oncogenesis or other tumor processing in a complicated microenvironment in lower respiratory tracts, further validated by a restricted proportion of functional cascades based on the KEGG pathways (**Figures 5C**, **S5C**). These results were also in accordance with various previous works that showed that the utilization of complex metabolites to induce local chronic inflammatory stimulation may be one of the dominant factors in microbial-mediated tumor development and progression (Hosseinkhani et al., 2021).

## Discussion

In this study, we aimed to address the compositional discrepancy between the microbial components detected in BALF samples obtained from healthy lung segments and tumor-burden lung segments in the same patient by electronic bronchoscopy mediated invasive sampling approach, focusing on adults with untreated lung cancer. Our findings confirmed niche specificity of microbiota in malignant lesions loaded segments and normal bronchial surface but indicated that the architecture of the bacterial communities in two types of different segments slightly differed with quite limited differential bacterial abundance, which might contribute to oncogenesis in a dynamic process. Of the intermediate metabolites of lipid metabolism detection, our observations collectively supported that the specific original microbiota related closely with the production and release of SCFAs in this cohort, recognizing that the dominance of a set of candidate species in tumor-burden lung segments might be the main genus of bacteria producing SCFAs, supporting that a single metabolite weakened the predictive value and that a combined model would be a priority. Additionally, our analyses by multiple approaches consistently found that microbiota willingly promotes oncogenesis by activating host cell signaling by microbial metabolites, including SCFAs. This finding might be opposite to those of previous studies, suggesting that this deserves experimental verifications and clinical analysis in detail.

Commensal microbial dysbiosis has been regarded as a primary carcinogenic factor to carcinogenesis and progression by bilateral interaction between microbiota and the host, including microbes in tumor-resident intracellular microbiota (Fu et al., 2022), intra-tumoral extracellular microbiota (Nejman et al., 2020), gut microbiome (Sepich-Poore et al., 2021), and those in localized microenvironment. Technically, the outburst of metagenomic sequencing dispelled the cloud overhead that the lower respiratory tract is sterile (Teague et al., 1981), accelerating the extensive explorations of lower respiratory tract microbiomes in lung carcinogenesis and malignant biological behaviors. Various studies have reported the frequent association of *Streptococcus*, *Staphylococcus*, *Pseudomonas*, and *Veillonella* in lung cancer (Fu et al., 2022), which could be altered dynamically by primary lesion types and progression, metastatic sites formation, and complications accompanied in clinical settings (Garg et al., 2017). In accordance with other microbe-mediated oncogenesis, the lower respiratory tract microbiome is also prone to trigger tumor initiation by inducing DNA damage, activating oncogenic and inflammatory pathways, breaking anti-tumor immunity balance, and most likely, releasing microbe-oriented cytotoxic metabolites (Sepich-Poore et al., 2021). Segal’s studies illustrated that the exposure of airway epithelial cells to tumor-associated microbes upregulated ERK and PI3K pathways by lower airway transcriptome in patients with cancer, possibly by activating IL-17 inflammatory phenotype (Tsay et al., 2018). In our study, we found that the abundance of *Campylobacter jejuni*, also detected by other groups (Canning et al., 2013; Zheng et al., 2021), shared a close connection with lung cancer, and several species were also sequenced in normal segments, which perhaps dressed up as probiotics in localized microenvironment. Unfortunately, as the same with other studies, we failed to demonstrate the specific oncogenic or anti-oncogenic roles of these diverse microbiota in lung cancer due to the lack of appropriate models *in vivo* and the complexity of microbial pathogenesis. Given that the majority of studies on lower respiratory tract microbiome concentrated on its potential relevance with lung cancer clinically (**Table S5**), in-depth studies are still needed to shed light on mechanical insights, owing to microbial compositional diversity and differential pathogenicity of lower respiratory tract microbiome.

SCFAs are mainly generated by non-digestive and fermentable carbohydrates from the gut microbiome, some of which can also be produced by host cells during normal cellular processes, performing as widely recognized protective metabolites in multiple cancer types. With total intestinal concentration exceeding 100mM, SCFAs released by the gut microbiome exert beneficial effects on gastrointestinal cancer and can also mediate tumoral inhibition of distant organs by large amounts of SCFA influx into the bloodstream via several gut axes (Liu et al., 2021). As to lung cancer, the lower respiratory tract microbiome should also be viewed as a vital source of SCFAs besides intestinal tracts, even if with a quite limited concentration, which is in line with the perspective that elevated SCFAs in the cancer group act as a sign of abnormal bacterial growth in the damaged lung (Dickson et al., 2015). Unfortunately, few studies have focused on the determination of respiratory microbiota-derived SCFAs in mediating lung cancer, which limits the understanding of their possible functions in the maintenance of respiratory immunity homeostasis. Based on the above, our study examined the concentration of SCFAs from BALF samples in different lung segments, finding that a slight difference of SCFAs was detected in tumor-burden lung segments compared to healthy segments, which was in agreement with previous findings (Yue et al., 2020). After excluding the detection errors induced by lavage liquids, machine learning profiles supported that the results that integrated prediction models of SCFA candidates, including VA, CA, and IBV, were more important compared to a single agent in lung cancer screening and diagnosis. It is still well-established that the lower respiratory tract microbiota is linked to lung cancer either directly via secreted SCFAs that stop the disease’s progression or by producing other substances on host cells that start metabolic reprogramming. However, more research is necessary to fully understand this association’s powerful mechanical effects.

As to the crucial prerequisite for revealing the compositional role of the microbiome, accurately measuring the low biomass microbiota in the lower airways is still challenging in the deep sequencing era (Huang and Boushey, 2015). Bacterial DNA density is at least 100 times lower in the lower respiratory tract than in the upper airways, compromising accuracy due to potential sampling and processing contamination (Dickson et al., 2017; Schneeberger et al., 2019). In this study, we standardized protected sampling of the lower respiratory tract to minimize artificial and systematic contamination, including homogeneous samples of ipsilateral lung segments from the same patient to reduce individual differences, strict aseptic technique processes and materials to restrict man-made interferences, and precise sequencing data of metagenomic and metabolic detection to lower confounding bias to a certain extent. Cohorts from the same center additionally ensured a uniform approach for operating processes from healthy segments to tumor-burden ones, as well as for electronic bronchoscope evaluation. Additionally, the simultaneous processing, storage, and testing of the two sets of samples reduced unanticipated growth and metabolic activity. Briefly, except for inevitable noises from collecting sequence, such as bronchoalveolar lavages in tumor-burden segments after those in healthy ones and microbiota compositional diversities in different lung segments in the same patient, the uniformity of sample collection and processing greatly reduces systematic errors and further guarantees the accuracy of a realistic composition of the lower respiratory tract microbiota and corresponding metabolism in lung cancer compared with healthy controls. Even though a degree of cross-contamination was inevitable, the confounding factors have been minimized in the design and actual implementation of this study.

Another issue that deserves additional attention is the causality between malignant lesion formation and microbial composition alteration in a spatiotemporal dynamic manner. Due to the abnormal outward proliferation that breaks through the basement membrane, the distal end of alien organisms in the airway was prone to be in a relatively hypoxic state (West, 1978), which may facilitate the proliferation of anaerobic bacteria and weaken the aerobic bacterial content accordingly. The dominant presence of specific bacterial genera, especially anaerobic or facultative anaerobic organisms, causes ripples throughout the tumor partly and even entirely via abnormal production of bacterial metabolites. This restrictive interaction makes tumors settle at a certain stage and forms a specific tumor microenvironment, which can be switched by perturbation of microbiota or rapid changes in tumor cell load. On the other hand, unrelenting nutrient transformation within the local microecological environment surrounding tumors inevitably contributes to competition between microorganisms and host cells, leading to dynamic changes in both species and quantity of microbial community. Although tumor cell-mediated nutritional deprivation undermines the energy supply of the microbial community, the slight variations induced by the imbalance of the microbiota in the lower respiratory tract still matter in tumor progression as a non-negligible biological point. According to our study sampling the microbiome and targeted metabolites at a restricted time, dynamic monitoring based on different stages of tumor progression still requires additional attention from large-scale examinations, which should aim to further uncover in detail microbial dysbiosis-mediated oncogenesis or vice versa.

Several limitations may shadow the outcomes of this study. First, restricted participants in a single center probably magnified the selective bias, leading to a distanced state from genuine microbial communities and metabolites in the lower respiratory tract with its densely packed low biomass. Additionally, due to successive sample collection from different lung segments in the same lung cancer patient and lack of negative control from healthy subjects and those with benign respiratory disease, BALFs were prone to be affected by operational sequence, inducing nuances of microbial composition and metabolic content. Furthermore, a complex composition of various microbiota-released metabolites detected from BALF in the lower respiratory tract was not distinguished in this study, which was liable to weaken the protective role of SCFAs. Finally, the dynamic interaction between the host and microbiota via metabolites makes it challenging to determine the actual source of these microbiota-oriented metabolites, leading to confounding bias in our data. *In vitro* experiments detaching from the whole dimmed the holistic influence on lung cancer, inspired by complicated microbial and microbe-host interactions.

## Conclusions

In our 28-participant-enrolled cohort, the lower respiratory tract microbiome and relative SCFAs detected in paired bronchoalveolar lavage fluids from normal lung segments and tumor-burden lung segments of the same patient were investigated. We found that different regions of the same patients’ lower respiratory tract microbiomes exhibit distinct signals. Furthermore, neither group’s SCFAs had any value as a single predictor, but combined analysis may be able to forecast the connection of SCFAs to oncogenesis. Additionally, by the production of specific metabolites, such as SCFAs, some microbial species in lung regions with tumor load were able to influence oncogenesis or serve as a predictor. Therefore, self-control studies of extended samples may be advantageous for future studies intended to clarify the preventative, diagnostic, and therapeutic significance of lower respiratory tract microbiota contributing to tumor blocking.

## Data availability statement

All data generated or analyzed in this study were oriented from a standardized clinical process and are included in this published article. Sequence data that support the findings of this study have been deposited in the NCBI Short Read Archive with the primary Bioproject accession code PRJNA991321 on the following link: https://www.ncbi.nlm.nih.gov/sra/PRJNA991321.

## Ethics statement

This research presented here has been performed in accordance with the Declaration of Helsinki and was approved by the Ethics Committee of the First Affiliated Hospital of the Air Force Medical University (#XJYY-LL-FJ-002). The patients included in this research have signed informed consent forms based on the voluntary principle before sample collection performance.

## Author contributions

YoZ: Data curation, Visualization, Writing – original draft. XC: Data curation, Formal Analysis, Resources, Writing – original draft. YW: Investigation, Methodology, Writing – original draft. LL: Formal Analysis, Project administration, Software, Writing – review & editing. QJ: Investigation, Validation, Writing – review & editing. YaZ: Methodology, Resources, Validation, Writing – review & editing. HX: Methodology, Validation, Writing – review & editing. FW: Methodology, Validation, Writing – review & editing. DQ: Resources, Software, Writing – original draft. XL: Formal Analysis, Visualization, Writing – review & editing. NC: Project administration, Resources, Writing – review & editing. WZ: Project administration, Software, Writing – review & editing. CZ: Formal Analysis, Validation, Writing – review & editing. KW: Funding acquisition, Investigation, Writing – review & editing. LL: Funding acquisition, Supervision, Writing – review & editing. JZ: Conceptualization, Funding acquisition, Supervision, Writing – review & editing.

## Supplementary material

Supplementary Figure 1 Workflow of study cohort enrollment with inclusion and exclusion criteria.

Supplementary Figure 2 Microbial composition and diversity comparison within each sample. **(A, B)** Relative abundance of the lower respiratory tract microbiota in the indicated groups at phylum and genus level, respectively. **(C, D)** 3D PCA and PCoA analysis plot of lower respiratory tract microbiome in normal and tumor burden lung segments. PCA, principal component analysis. PCoA, principal coordinates analysis. **(E, F)** Relative abundance of the lower respiratory tract microbiota in each sample at phylum and genus level, respectively. **(G)** CIRCOS plot of taxonomic abundance among each samples.

Supplementary Figure 3 Differential microbiota comparison and co-occurrence network. **(A)** Box plots of relative abundance within indicated significant differential microbiota among both groups. **(B)** Microbial co-occurrence network of different candidates from Figure S3A. Each node represents a species and edges correspond to significant species-species associations. The size of each node is proportional to the mean relative abundance at the phylum level. The 95% credible criteria were used to assess significance, and estimated correlations were then filtered with the correlation coefficient≥0.4 in a line thickness-dependent format. Color labels are marked by orange (positive correlation) and blue (negative correlation), respectively.

Supplementary Figure 4 Key parameter supplementation of LASSO and Random Forest. **(A)** Profiles of LASSO regression regarding partial likelihood deviance and misclassification error. The lines indicate the 95% confidence interval of the regression, and the dotted line represents the optimal number of variables. **(B)** Representative OOB error estimate based on random forest among indicated groups. OOB, out-of-bag.

Supplementary Figure 5 Relative abundance of KEGG pathways in both groups. **(A, B)** Relative abundance of KEGG levels 1 and 2 within both groups. **(C)** Distribution diagram of the KEGG pathways based on LDA score among indicated groups.

## References

[B1] Asnicar F. Berry S. E. Valdes A. M. Nguyen L. H. Piccinno G. Drew D. A.. (2021). Microbiome connections with host metabolism and habitual diet from 1,098 deeply phenotyped individuals. Nat. Med. 27, 321–332. doi: 10.1038/s41591-020-01183-8 33432175 PMC8353542

[B2] Bhatt A. P. Redinbo M. R. Bultman S. J. (2017). The role of the microbiome in cancer development and therapy. CA Cancer J. Clin. 67, 326–344. doi: 10.3322/caac.21398 28481406 PMC5530583

[B3] Canning C. Sun S. Ji X. Gupta S. Zhou K. (2013). Antibacterial and cytotoxic activity of isoprenylated coumarin mammea A/AA isolated from Mammea africana. J. Ethnopharmacol. 147, 259–262. doi: 10.1016/j.jep.2013.02.026 23466248

[B4] Cullin N. Azevedo Antunes C. Straussman R. Stein-Thoeringer C. K. Elinav E. (2021). Microbiome and cancer. Cancer Cell 39, 1317–1341. doi: 10.1016/j.ccell.2021.08.006 34506740

[B5] Dickson R. P. Erb-Downward J. R. Freeman C. M. Mccloskey L. Falkowski N. R. Huffnagle G. B.. (2017). Bacterial topography of the healthy human lower respiratory tract. mBio 8 (1), e02287–16. doi: 10.1128/mBio.02287-16 28196961 PMC5312084

[B6] Dickson R. P. Erb-Downward J. R. Huffnagle G. B. (2015). Homeostasis and its disruption in the lung microbiome. Am. J. Physiol. Lung Cell Mol. Physiol. 309, L1047–L1055. doi: 10.1152/ajplung.00279.2015 26432870 PMC4652146

[B7] Dohlman A. B. Klug J. Mesko M. Gao I. H. Lipkin S. M. Shen X.. (2022). A pan-cancer mycobiome analysis reveals fungal involvement in gastrointestinal and lung tumors. Cell 185, 3807–3822.e3812. doi: 10.1016/j.cell.2022.09.015 36179671 PMC9564002

[B8] Dong Q. Chen E. S. Zhao C. Jin C. (2021). Host-microbiome interaction in lung cancer. Front. Immunol. 12, 679829. doi: 10.3389/fimmu.2021.679829 34108973 PMC8183378

[B9] Drengenes C. Wiker H. G. Kalananthan T. Nordeide E. Eagan T. M. L. Nielsen R. (2019). Laboratory contamination in airway microbiome studies. BMC Microbiol. 19, 187. doi: 10.1186/s12866-019-1560-1 31412780 PMC6694601

[B10] Fromentin M. Ricard J. D. Roux D. (2021). Respiratory microbiome in mechanically ventilated patients: a narrative review. Intensive Care Med. 47, 292–306. doi: 10.1007/s00134-020-06338-2 33559707 PMC7871139

[B11] Fu A. Yao B. Dong T. Chen Y. Yao J. Liu Y.. (2022). Tumor-resident intracellular microbiota promotes metastatic colonization in breast cancer. Cell 185, 1356–1372.e1326. doi: 10.1016/j.cell.2022.02.027 35395179

[B12] Garg N. Wang M. Hyde E. Da Silva R. R. Melnik A. V. Protsyuk I.. (2017). Three-dimensional microbiome and metabolome cartography of a diseased human lung. Cell Host Microbe 22, 705–716.e704. doi: 10.1016/j.chom.2017.10.001 29056429 PMC6267898

[B13] Glendinning L. Collie D. Wright S. Rutherford K. M. D. Mclachlan G. (2017). Comparing microbiotas in the upper aerodigestive and lower respiratory tracts of lambs. Microbiome 5, 145. doi: 10.1186/s40168-017-0364-5 29078799 PMC5658956

[B14] Hosseinkhani F. Heinken A. Thiele I. Lindenburg P. W. Harms A. C. Hankemeier T. (2021). The contribution of gut bacterial metabolites in the human immune signaling pathway of non-communicable diseases. Gut Microbes 13, 1–22. doi: 10.1080/19490976.2021.1882927 PMC7899087 33590776

[B15] Huang Y. J. Boushey H. A. (2015). The sputum microbiome in chronic obstructive pulmonary disease exacerbations. Ann. Am. Thorac. Soc. 12 Suppl 2, S176–S180. doi: 10.1513/AnnalsATS.201506-319AW 26595736 PMC4722839

[B16] Jin Y. Y. Shi Z. Q. Chang W. Q. Guo L. X. Zhou J. L. Liu J. Q.. (2018). A chemical derivatization based UHPLC-LTQ-Orbitrap mass spectrometry method for accurate quantification of short-chain fatty acids in bronchoalveolar lavage fluid of asthma mice. J. Pharm. BioMed. Anal. 161, 336–343. doi: 10.1016/j.jpba.2018.08.057 30199808

[B17] Kim M. H. Kang S. G. Park J. H. Yanagisawa M. Kim C. H. (2013). Short-chain fatty acids activate GPR41 and GPR43 on intestinal epithelial cells to promote inflammatory responses in mice. Gastroenterology 145, 396–406.e391-310. doi: 10.1053/j.gastro.2013.04.056 23665276

[B18] Kim M. Qie Y. Park J. Kim C. H. (2016). Gut microbial metabolites fuel host antibody responses. Cell Host Microbe 20, 202–214. doi: 10.1016/j.chom.2016.07.001 27476413 PMC4982788

[B19] Kurian S. M. Gordon S. Barrick B. Dadlani M. N. Fanelli B. Cornell J. B.. (2020). Feasibility and comparison study of fecal sample collection methods in healthy volunteers and solid organ transplant recipients using 16S rRNA and metagenomics approaches. Biopreserv. Biobank 18, 425–440. doi: 10.1089/bio.2020.0032 32833508

[B20] Lamoureux C. Surgers L. Fihman V. Gricourt G. Demontant V. Trawinski E.. (2022). Prospective comparison between shotgun metagenomics and sanger sequencing of the 16S rRNA gene for the etiological diagnosis of infections. Front. Microbiol. 13, 761873. doi: 10.3389/fmicb.2022.761873 35464955 PMC9020828

[B21] Lanaspa M. Bassat Q. Medeiros M. M. Muñoz-Almagro C. (2017). Respiratory microbiota and lower respiratory tract disease. Expert Rev. Anti Infect. Ther. 15, 703–711. doi: 10.1080/14787210.2017.1349609 28661199

[B22] Liu F. Li J. Guan Y. Lou Y. Chen H. Xu M.. (2019). Dysbiosis of the gut microbiome is associated with tumor biomarkers in lung cancer. Int. J. Biol. Sci. 15, 2381–2392. doi: 10.7150/ijbs.35980 31595156 PMC6775324

[B23] Liu Q. Tian X. Maruyama D. Arjomandi M. Prakash A. (2021). Lung immune tone via gut-lung axis: gut-derived LPS and short-chain fatty acids’ immunometabolic regulation of lung IL-1β, FFAR2, and FFAR3 expression. Am. J. Physiol. Lung Cell Mol. Physiol. 321, L65–l78. doi: 10.1152/ajplung.00421.2020 33851870 PMC8321849

[B24] Man W. H. De Steenhuijsen Piters W. A. Bogaert D. (2017). The microbiota of the respiratory tract: gatekeeper to respiratory health. Nat. Rev. Microbiol. 15, 259–270. doi: 10.1038/nrmicro.2017.14 28316330 PMC7097736

[B25] Matsushita M. Fujita K. Hayashi T. Kayama H. Motooka D. Hase H.. (2021). Gut microbiota-derived short-chain fatty acids promote prostate cancer growth via IGF1 signaling. Cancer Res. 81, 4014–4026. doi: 10.1158/0008-5472.CAN-20-4090 34039634

[B26] Mirzaei R. Afaghi A. Babakhani S. Sohrabi M. R. Hosseini-Fard S. R. Babolhavaeji K.. (2021). Role of microbiota-derived short-chain fatty acids in cancer development and prevention. BioMed. Pharmacother. 139, 111619. doi: 10.1016/j.biopha.2021.111619 33906079

[B27] Nejman D. Livyatan I. Fuks G. Gavert N. Zwang Y. Geller L. T.. (2020). The human tumor microbiome is composed of tumor type-specific intracellular bacteria. Science 368, 973–980. doi: 10.1126/science.aay9189 32467386 PMC7757858

[B28] Patnaik S. K. Cortes E. G. Kannisto E. D. Punnanitinont A. Dhillon S. S. Liu S.. (2021). Lower airway bacterial microbiome may influence recurrence after resection of early-stage non-small cell lung cancer. J. Thorac. Cardiovasc. Surg. 161, 419–429.e416. doi: 10.1016/j.jtcvs.2020.01.104 32340803

[B29] Qin J. Li R. Raes J. Arumugam M. Burgdorf K. S. Manichanh C.. (2010). A human gut microbial gene catalogue established by metagenomic sequencing. Nature 464, 59–65. doi: 10.1038/nature08821 20203603 PMC3779803

[B30] Routy B. Le Chatelier E. Derosa L. Duong C. P. M. Alou M. T. Daillère R.. (2018). Gut microbiome influences efficacy of PD-1-based immunotherapy against epithelial tumors. Science 359, 91–97. doi: 10.1126/science.aan3706 29097494

[B31] Schneeberger P. H. H. Prescod J. Levy L. Hwang D. Martinu T. Coburn B. (2019). Microbiota analysis optimization for human bronchoalveolar lavage fluid. Microbiome 7, 141. doi: 10.1186/s40168-019-0755-x 31665066 PMC6821041

[B32] Sepich-Poore G. D. Zitvogel L. Straussman R. Hasty J. Wargo J. A. Knight R. (2021). The microbiome and human cancer. Science 371 (6536), eabc4552. doi: 10.1126/science.abc4552 33766858 PMC8767999

[B33] Shen H. Lu Z. Xu Z. Chen Z. Shen Z. (2017). Associations among dietary non-fiber carbohydrate, ruminal microbiota and epithelium G-protein-coupled receptor, and histone deacetylase regulations in goats. Microbiome 5, 123. doi: 10.1186/s40168-017-0341-z 28927467 PMC5606034

[B34] Singh N. Vats A. Sharma A. Arora A. Kumar A. (2017). The development of lower respiratory tract microbiome in mice. Microbiome 5, 61. doi: 10.1186/s40168-017-0277-3 28637485 PMC5479047

[B35] Sivaprakasam S. Prasad P. D. Singh N. (2016). Benefits of short-chain fatty acids and their receptors in inflammation and carcinogenesis. Pharmacol. Ther. 164, 144–151. doi: 10.1016/j.pharmthera.2016.04.007 27113407 PMC4942363

[B36] Teague R. B. Wallace R. J. Jr. Awe R. J. (1981). The use of quantitative sterile brush culture and gram stain analysis in the diagnosis of lower respiratory tract infection. Chest 79, 157–161. doi: 10.1378/chest.79.2.157 6161757

[B37] Trompette A. Gollwitzer E. S. Yadava K. Sichelstiel A. K. Sprenger N. Ngom-Bru C.. (2014). Gut microbiota metabolism of dietary fiber influences allergic airway disease and hematopoiesis. Nat. Med. 20, 159–166. doi: 10.1038/nm.3444 24390308

[B38] Tsang N. N. Y. So H. C. Ng K. Y. Cowling B. J. Leung G. M. Ip D. K. M. (2021). Diagnostic performance of different sampling approaches for SARS-CoV-2 RT-PCR testing: a systematic review and meta-analysis. Lancet Infect. Dis. 21, 1233–1245. doi: 10.1016/S1473-3099(21)00146-8 33857405 PMC8041361

[B39] Tsay J. J. Wu B. G. Badri M. H. Clemente J. C. Shen N. Meyn P.. (2018). Airway microbiota is associated with upregulation of the PI3K pathway in lung cancer. Am. J. Respir. Crit. Care Med. 198, 1188–1198. doi: 10.1164/rccm.201710-2118OC 29864375 PMC6221574

[B40] Ubachs J. Ziemons J. Soons Z. Aarnoutse R. Van Dijk D. P. J. Penders J.. (2021). Gut microbiota and short-chain fatty acid alterations in cachectic cancer patients. J. Cachexia Sarcopenia Muscle 12, 2007–2021. doi: 10.1002/jcsm.12804 34609073 PMC8718054

[B41] Van Der Hee B. Wells J. M. (2021). Microbial regulation of host physiology by short-chain fatty acids. Trends Microbiol. 29, 700–712. doi: 10.1016/j.tim.2021.02.001 33674141

[B42] West J. B. (1978). Regional differences in the lung. Chest 74, 426–437. doi: 10.1378/chest.74.4.426 699656

[B43] Yang L. Li A. Wang Y. Zhang Y. (2023). Intratumoral microbiota: roles in cancer initiation, development and therapeutic efficacy. Signal Transduct. Target Ther. 8, 35. doi: 10.1038/s41392-022-01304-4 36646684 PMC9842669

[B44] Yue M. Kim J. H. Evans C. R. Kachman M. Erb-Downward J. R. D’souza J.. (2020). Measurement of short-chain fatty acids in respiratory samples: keep your assay above the water line. Am. J. Respir. Crit. Care Med. 202, 610–612. doi: 10.1164/rccm.201909-1840LE PMC7427402 32795141

[B45] Zheng L. Sun R. Zhu Y. Li Z. She X. Jian X.. (2021). Lung microbiome alterations in NSCLC patients. Sci. Rep. 11, 11736. doi: 10.1038/s41598-021-91195-2 34083661 PMC8175694

[B46] Zitvogel L. Kroemer G. (2021). Lower airway dysbiosis exacerbates lung cancer. Cancer Discov 11, 224–226. doi: 10.1158/2159-8290.CD-20-1641 33531424

[B47] Zou J. Chassaing B. Singh V. Pellizzon M. Ricci M. Fythe M. D.. (2018). Fiber-mediated nourishment of gut microbiota protects against diet-induced obesity by restoring IL-22-mediated colonic health. Cell Host Microbe 23, 41–53.e44. doi: 10.1016/j.chom.2017.11.003 29276170 PMC6005180

