# Supplementary figures and images for: Alterations of lower respiratory tract microbiome and short-chain fatty acids in different segments in lung cancer: a multiomics analysis
Source: Front Cell Infect Microbiol. 2023 Oct 16;13:1261284. doi: 10.3389/fcimb.2023.1261284 (PMC10617678; doi:10.3389/fcimb.2023.1261284)

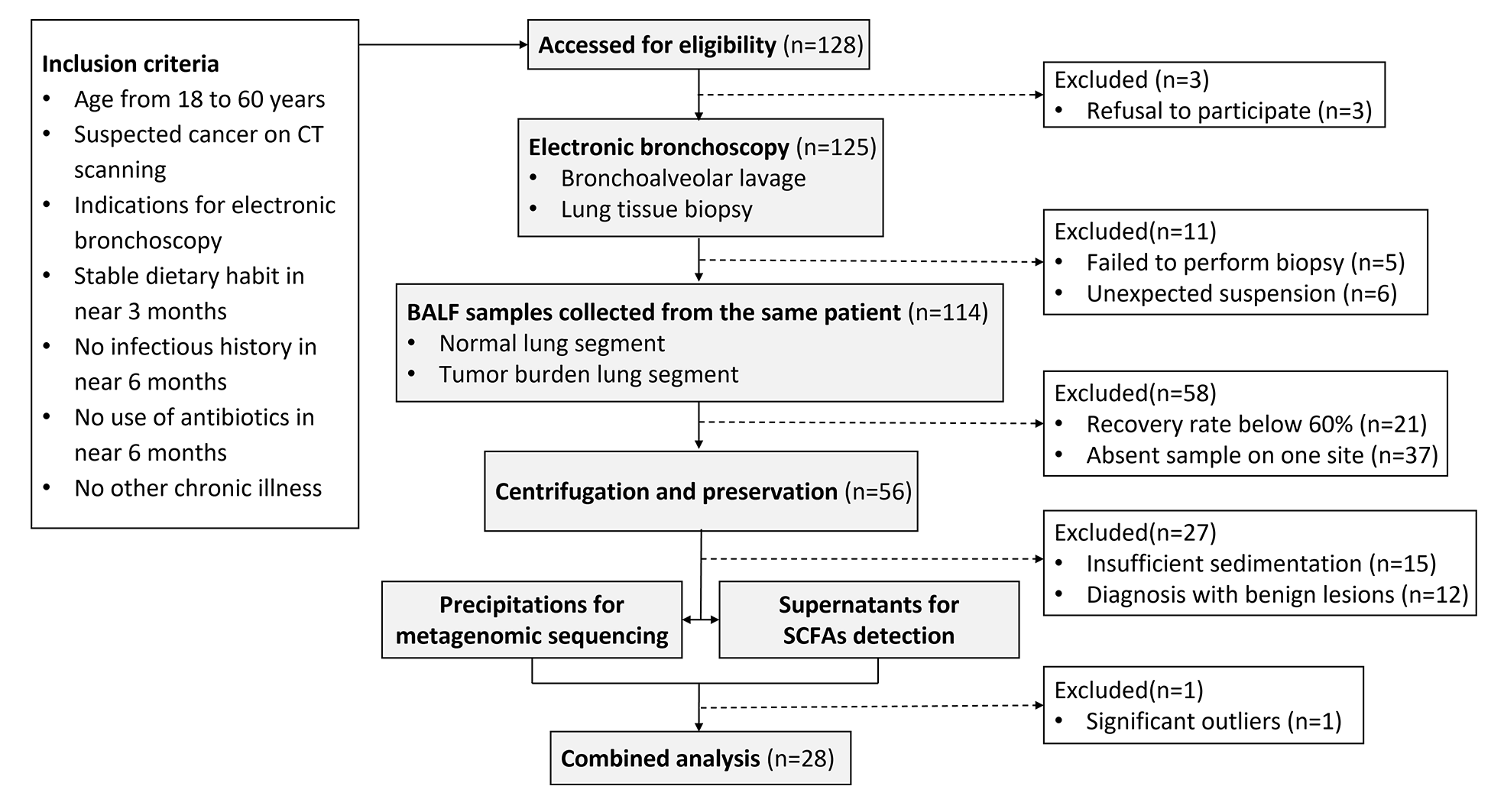

Supplement: Supplementary Figure 1 — Workflow of study cohort enrollment with inclusion and exclusion criteria. [file Image_1.tif]

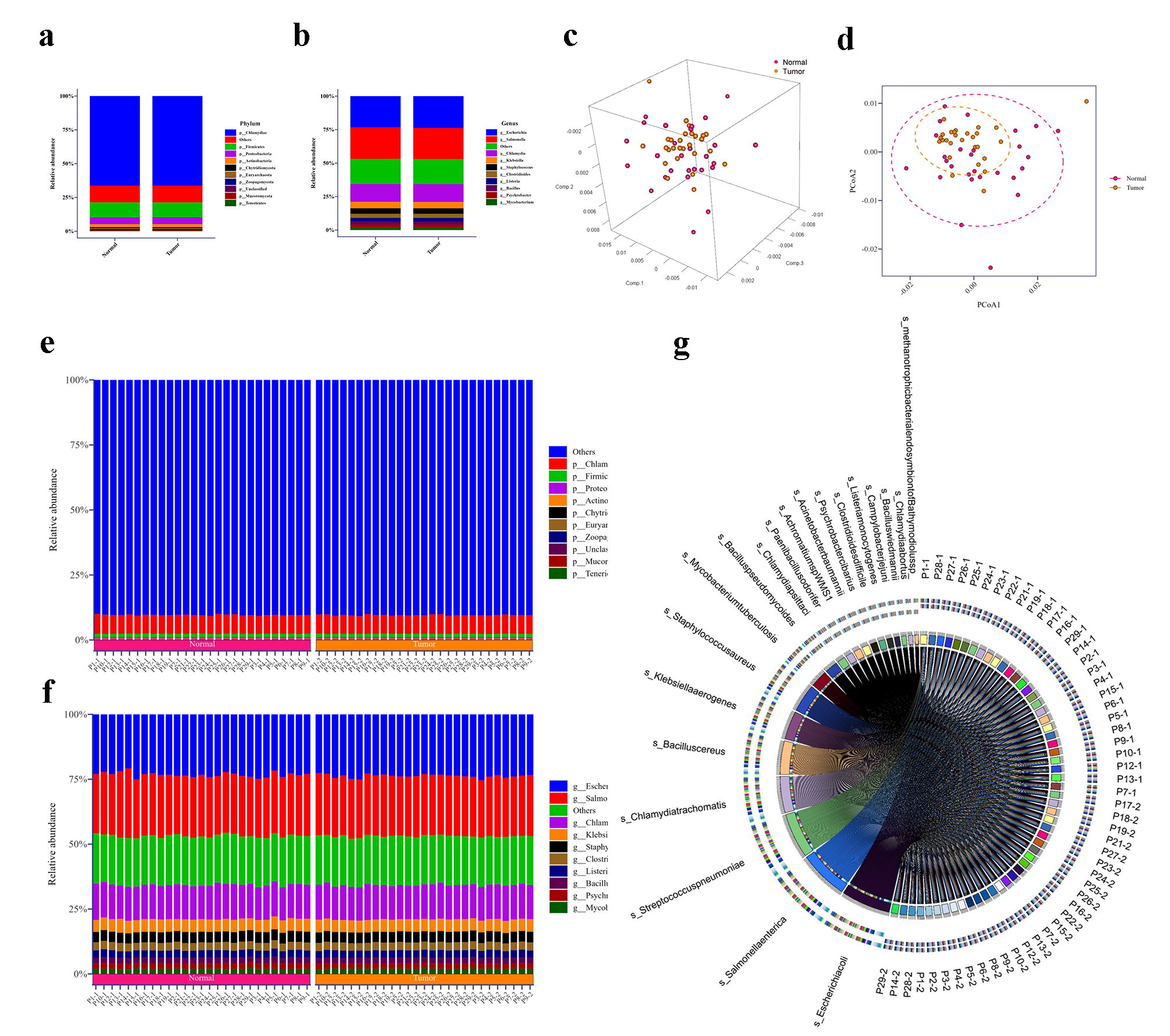

Supplement: Supplementary Figure 2 — Microbial composition and diversity comparison within each sample. (A, B) Relative abundance of the lower respiratory tract microbiota in the indicated groups at phylum and genus level, respectively. (C, D) 3D PCA and PCoA analysis plot of lower respiratory tract microbiome in normal and tumor burden lung segments. PCA, principal component analysis. PCoA, principal coordinates analysis. (E, F) Relative abundance of the lower respiratory tract microbiota in each sample at phylum and genus level, respectively. (G) CIRCOS plot of taxonomic abundance among each samples. [file Image_2.tif]

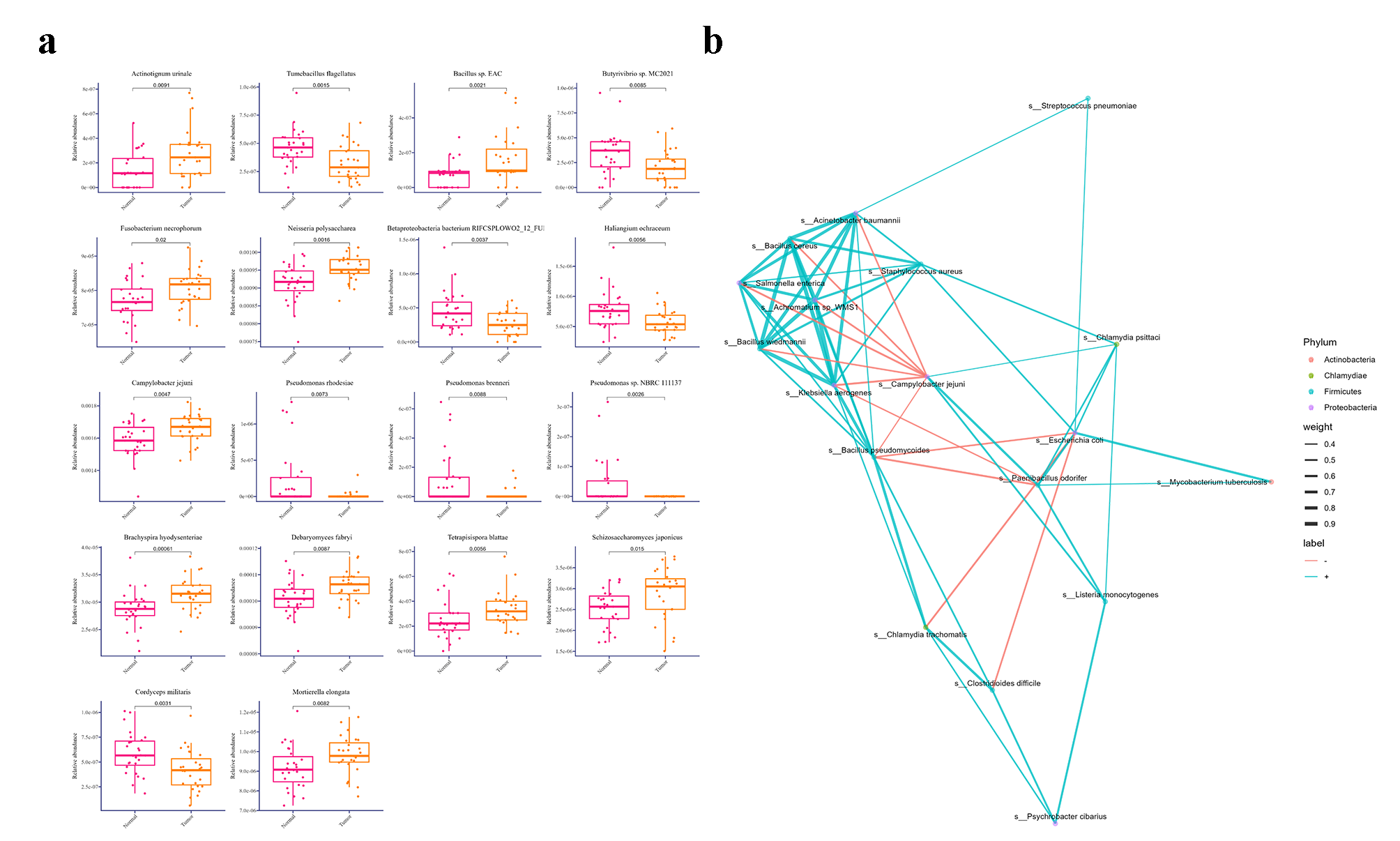

Supplement: Supplementary Figure 3 — Differential microbiota comparison and co-occurrence network. (A) Box plots of relative abundance within indicated significant differential microbiota among both groups. (B) Microbial co-occurrence network of different candidates from Figure S3A. Each node represents a species and edges correspond to significant species-species associations. The size of each node is proportional to the mean relative abundance at the phylum level. The 95% credible criteria were used to assess significance, and estimated correlations were then filtered with the correlation coefficient≥0.4 in a line thickness-dependent format. Color labels are marked by orange (positive correlation) and blue (negative correlation), respectively. [file Image_3.tif]

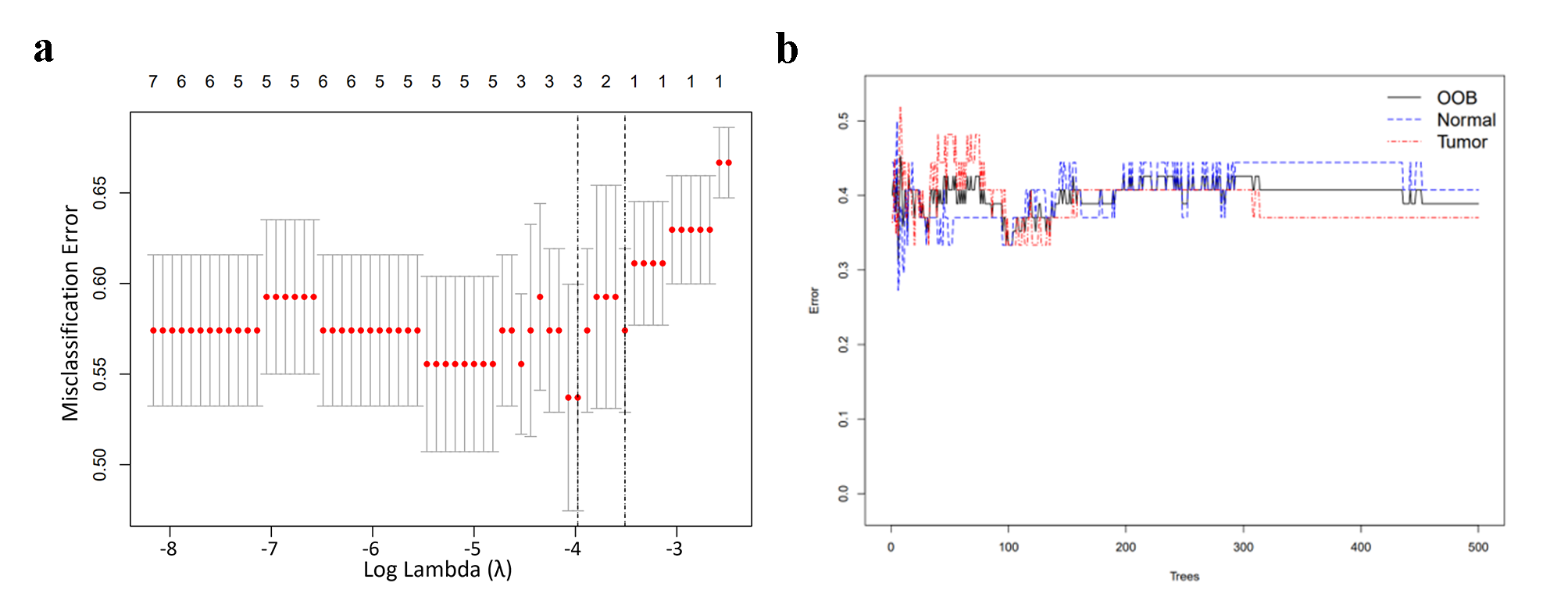

Supplement: Supplementary Figure 4 — Key parameter supplementation of LASSO and Random Forest. (A) Profiles of LASSO regression regarding partial likelihood deviance and misclassification error. The lines indicate the 95% confidence interval of the regression, and the dotted line represents the optimal number of variables. (B) Representative OOB error estimate based on random forest among indicated groups. OOB, out-of-bag. [file Image_4.tif]

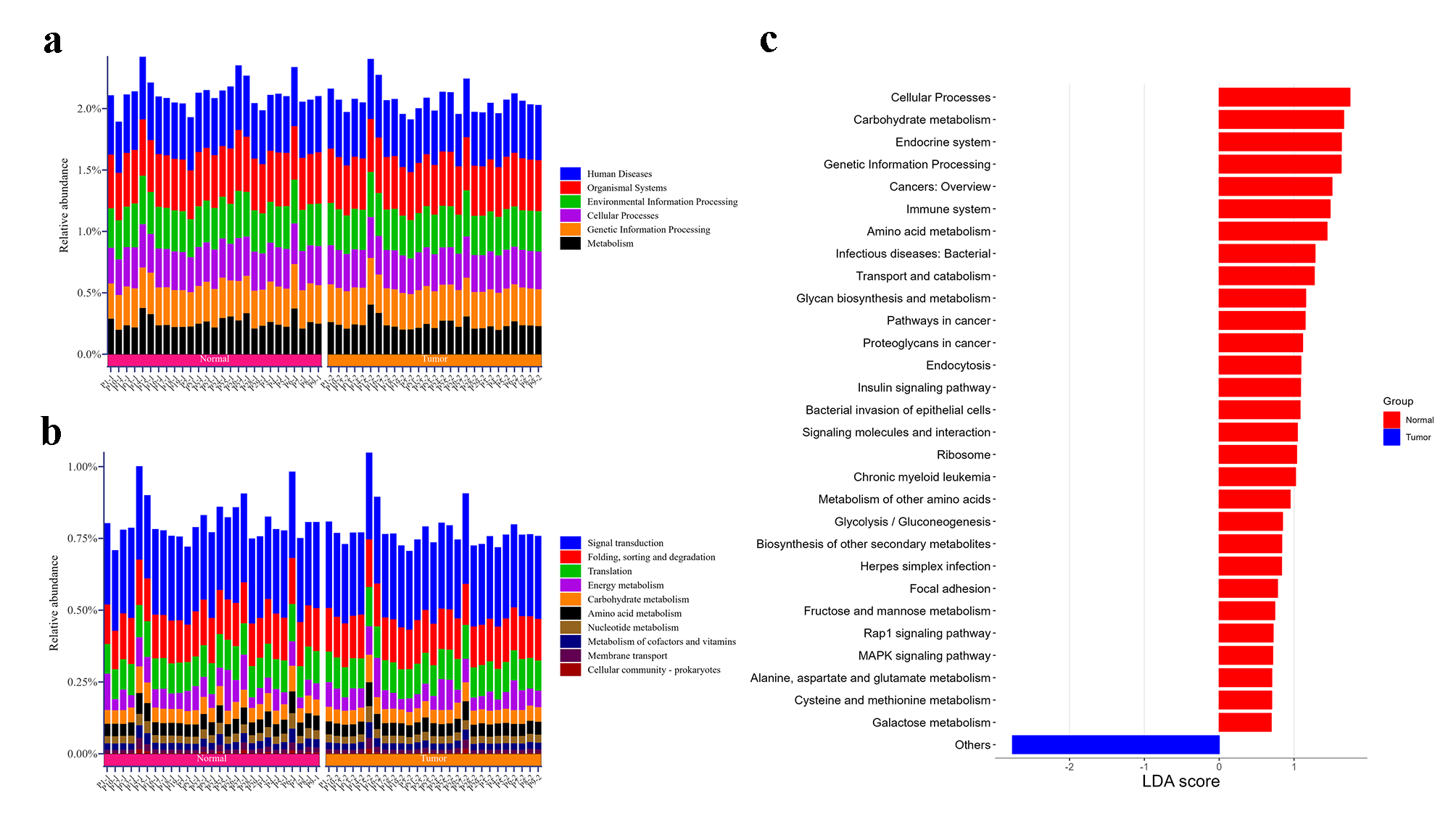

Supplement: Supplementary Figure 5 — Relative abundance of KEGG pathways in both groups. (A, B) Relative abundance of KEGG levels 1 and 2 within both groups. (C) Distribution diagram of the KEGG pathways based on LDA score among indicated groups. [file Image_5.tif]
